# National Survey on bladder and bowel dysfunctions in Autism Spectrum Disorder population

**DOI:** 10.3389/fpsyt.2024.1140113

**Published:** 2024-03-11

**Authors:** Marilena Gubbiotti, Matteo Balzarro, Leonardo Zoccante, Gianfranco Di Gennaro, Moreno Marchiafava, Chiara Bedetti, Emanuele Rubilotta

**Affiliations:** ^1^ Department of Urology, San Donato Hospital, Arezzo, Italy; ^2^ Department of Urology, A.O.U.I. Verona University, Verona, Italy; ^3^ Department of Child Neuropsychiatry, A.O.U.I. Verona University, Verona, Italy; ^4^ Department of Health Sciences, “Magna Graecia” University of Catanzaro, Catanzaro, Italy; ^5^ Department of Mental Health and Pathological Addictions, ASL Roma 5, Roma, Italy; ^6^ Stroke Unite/Neurology, Città di Castello Hospital, Perugia, Italy

**Keywords:** autism spectrum disorder, urinary dysfunction, urinary tract symptoms, survey, bladder and bowel dysfunction

## Abstract

**Introduction:**

To evaluate lower urinary tract symptoms (LUTS) and bowel disorders in a population of young subjects with autism spectrum disorder (ADS) by a national survey and to assess the relationship between the occurrence, frequency, and type of LUTS and the severity of behavioral and neuropsychiatric characteristics.

**Materials and methods:**

A survey on LUTS and bowel disorders in the ASD population was sent by mail and social media through the main Italian Associations of ASD between February and September 2022. The correlation between LUTS and ASD severity was also assessed.

**Results:**

The survey was completed by 502 subjects with a mean age of 16.6 years ± 10 years: male participants were 413 (mean age: 16.5 years ± 9.8 years), while female participants 89 (mean age: 17.2 years ± 10.9 years). ADS severity was found low in 29.9%, moderate in 27.1%, and severe in 43%. LUTS were reported by 77.1%, storage symptoms in 51.4%, and voiding symptoms in 60.6%. Urinary incontinence was reported by 12.5%. Enuresis was reported by 14.3% (72/502) of the respondents: primary enuresis in 70.8% (51/72), secondary in the remaining. Pads were used by 40 subjects with a median of 2.9 pads/day (range, 0–8). A toilet training program was performed by 61 of the respondents, with satisfactory results in 40/61 (65.6%). A significant correlation was found between greater ASD severity and higher LUTS rates. The mean VAS score on the impact of LUTS on family relationships was 2 ± 2.9. Regular bowel function was reported by 57.4% (288/502) of the respondents, while increased daily defecations were present in 11.2% (56/502), constipation in 31.5% (158/502), and fecal incontinence in 7.9% (40/502).

**Conclusion:**

This survey demonstrated that LUTS are very common in the young ASD population and that the prevalence of urinary symptoms is related to higher severity of the ASD condition. Bowel disorders are often associated with urinary symptoms and dysfunctions. Urologists should be aware of the frequent occurrence of urological disorders and symptoms in individuals with ASD and should be involved in their clinical management in a multidisciplinary team that cares for these people.

## Introduction

Lower urinary tract symptoms (LUTS) are very common in both non-neurological and neurological populations, with rates reported up to 70% (1, 2). However, few data exist on LUTS in subjects with autism spectrum disorder (ASD) (3–5). In ASD, psychiatric and developmental alterations, as well as affective and attention disturbances, are very common and truly investigated (6–9). Conversely, only a few studies have demonstrated the high prevalence of vesico-sphincter and bowel dysfunctions in ASD pediatric populations (10, 11), while even fewer reports have been published on adolescents/adults (3, 12). To date, the most commonly described urinary disorders are overactive bladder and urinary incontinence (up to 87%), nocturnal enuresis (up to 72%), urgency (up to 31%), dysfunctional voiding (up to 15%), and fecal incontinence (up to 33%) (4, 12, 13). However, data were derived from a few studies with low sample sizes due to the objective difficulty of obtaining information. Consequently, the real need for urological help in this population is still unclear.

The aim of this study was to evaluate LUTS and bowel disorders in a large population of young ADS subjects through a national survey. A second endpoint was to assess the relationship between the occurrence, frequency, and type of LUTS and the severity of behavioral and neuropsychiatric characteristics.

## Materials and methods

A survey on LUTS and bowel disorders in the ASD population was sent by mail and social media (WhatsApp and Facebook) through the main Italian Associations of ASD between February and September 2022. This survey was developed by a panel of expert clinicians, composed of urologists (E.R., M.G.), a neurologist (C.B), a psychiatrist (M.M.), and a child neuropsychiatrist (L.Z.). The survey, named “Bladder and Bowel Dysfunctions in Autism-Questionnaire, BBDA-Q” (Appendix), was validated in Italian language and consisted of 22 questions, divided into three items: (i) five demographic questions; (ii) three questions evaluating the severity of ASD and need of aid by caregivers; and (iii) 14 questions on BBD.

Based on self-sufficiency in the daily activities and neurodevelopment condition, impairments in verbal or nonverbal communication and social interaction, and repetitive and restricted behaviors, the severity of the ASD condition was stratified into level 1 (low need of support: mild condition), level 2 need of support: moderate condition), and level 3 (severe need of support: severe condition).

Storage and voiding LUTS investigated were as follows: increased or reduced urinary frequency (how many micturition per day), urgency (yes/no), slow stream (yes/no), hesitancy (yes/no), and straining (yes/no). Urinary incontinence (daily and nocturnal) and nocturnal enuresis (yes/no) were also assessed. The use of pads (yes/no; No. of pads/day; how many times) and performing toilet training (yes/no; with poor/good results), were evaluated. Symptomatic subjects were considered in cases where at least one LUTS was reported.

The occurrence of sleep disorders, gastrointestinal disorders, urinary tract infections, immune system abnormalities/allergies/cutaneous diseases, and movement disabilities were also investigated.

The prevalence of BBD and its correlation with the level of ASD severity were assessed. A visual analogic scale (VAS) was used to assess the influence of LUTS on family relationships: 0 was *no influence*, and 10 was an *extremely negative influence*.

ASD subjects could be helped in the completion of the BBDA-Q by caregivers. Due to the type of ASD condition, validated LUTS questionnaires could not be used because they clearly did not fit the population we were evaluating. The Local Ethics Committee for Clinical Trials (CESC) determined that approval for this investigation was unnecessary since it only involved a survey on the usual voiding behaviors and no treatments were administered. This research was registered in the clinical audit in our hospital. CESC only required that the anonymity of the participants be guaranteed.

All questionnaires were anonymous; we just asked to include the gender and the age. Informed consent was asked and obtained to include data.

### Statistical analysis

Continuous variables were described by mean and standard deviation if normally distributed, or by median and interquartile range in cases of skewness. Counts and percentages were used for categorical variables. The prevalence of the following main outcomes, enuresis, urinary incontinence, urinary frequency, urinary urgency, and defecation abnormality was calculated together with the 95% confidence interval.

Differences between continuous variables were investigated using *t*-tests or Mann–Whitney tests, depending on the shape of the probability distribution. Categorical variables were investigated by equality tests for independent proportions. For each outcome, the relationship between age group and ASD severity was investigated using binomial logistic regression models. Analyses were conducted stratified by gender.

The statistical significance level was set at 5%. No missing data treatment strategy was preplanned. Q-square and ANOVA tests were also used. The analyses were conducted using STATA software version 17.1.

Psychometric analysis: the content validity was evaluated by a panel of clinicians and experts in the field of ASD.

## Results

The study sample consisted of 502 subjects, including 413 boys (82.2%) and 89 girls (17.7%). We obtained 61% of answers by social media and 39% by mail. The median age of girls was 15 years (IQR: 8–25 years), while the median age of boys was also 15 years but with a lower dispersion (IQR: 9–21 years). The difference was not statistically significant (Mann–Whitney test: *p* = 0.788). Overall collected data are described in **Table 1**. Subjects’ mean age was 16.6 years ± 10 years. The vast majority of respondents were boys (413) while girls were 98.

**Table 1:** Prevalence of urinary incontinence, enuresis, urinary urgency, change in urinary frequency, and altered frequency of defecation stratified according to gender, age group, and ASD levels.

Urinary incontinence	Odds ratio	95% CI		p	Enuresis	Odds ratio	95% CI		p
Males					Males				
Severity (ref: low)					Severity (ref: low)				
mild ASD	2.49	0.62	9.95	0.197	mild ASD	2.53	0.75	8.55	0.136
severe ASD	9.07	2.71	30.43	0.000	severe ASD	8.26	2.80	24.39	0.000
Age (ref: <6 years)					Age (ref: <6 years)				
6-12	0.45	0.15	1.34	0.151	6-12	0.21	0.08	0.55	0.002
13-18	0.29	0.09	0.97	0.044	13-18	0.13	0.05	0.39	0.000
>18	0.25	0.08	0.81	0.021	>18	0.07	0.02	0.22	0.000
Females					Females				
Severity (ref: low)					Severity (ref: low)				
mild ASD	0.87	0.11	6.89	0.895	mild ASD	1.04	0.03	inf	0.136
severe ASD	6.78	1.37	33.58	0.019	severe ASD	26.95	4.20	inf	0.000
Age (ref: <6 years)					Age (ref: <6 years)				
6-12	0.86	0.12	6.34	0.880	6-12	1.66	0.09	111.09	0.002
13-18	0.40	0.04	3.54	0.407	13-18	1.59	0.75	115.84	0.000
>18	0.68	0.09	4.90	0.701	>18	2.44	0.14	159.66	0.000
Urinary urgency	Odds ratio	95% CI		p	Change in urinary frequency	Odds ratio	95% CI		p
Males					Males				
Severity (ref: low)					Severity (ref: low)				
mild ASD	1.11	0.61	2.00	0.735	mild ASD	1.69	0.89	3.23	0.111
severe ASD	1.48	0.88	2.50	0.140	severe ASD	2.69	1.52	4.77	0.001
Age (ref: <6 years)					Age (ref: <6 years)				
6-12	0.46	0.19	1.09	0.078	6-12	0.55	0.22	1.35	0.193
13-18	0.41	0.17	1.02	0.054	13-18	0.62	0.25	1.56	0.311
>18	0.36	0.15	0.87	0.024	>18	0.45	0.18	1.12	0.087
Females					Females				
Severity (ref: low)					Severity (ref: low)				
mild ASD	3.34	0.92	12.11	0.067	mild ASD	0.79	0.13	4.64	0.792
severe ASD	2.55	0.77	8.40	0.125	severe ASD	2.51	0.60	10.59	0.210
Age (ref: <6 years)					Age (ref: <6 years)				
6-12	2.91	0.48	17.72	0.248	6-12	1.19	0.19	7.65	0.854
13-18	1.10	0.16	7.63	0.920	13-18	0.64	0.09	4.69	0.657
>18	2.10	0.35	12.47	0.415	>18	0.26	0.03	2.02	0.199
Altered frequency of defecation	Odds ratio	[95% conf. interval]		p					
Males									
Severity (ref: low)									
mild ASD	0.78	0.46	1.33	0.363					
severe ASD	0.67	0.42	1.07	0.091					
Age (ref: <6 years)									
6-12	1.21	0.51	2.84	0.665					
13-18	1.61	0.67	3.87	0.291					
>18	1.54	0.65	3.64	0.322					
									
Females									
Severity (ref: low)									
mild ASD	0.16	0.04	0.61	0.008					
severe ASD	0.18	0.05	0.65	0.009					
									
Age (ref: <6 years)									
6-12	1.09	0.20	6.02	0.921					
13-18	0.76	0.13	4.56	0.767					
>18	0.64	0.12	3.42	0.602					

According to ADS severity levels, subjects were stratified as follows: 150 (29.9%) respondents in level 1, 136 (27.1%) in level 2, and 216 (43%) in level 3.

Associated symptoms were sleep disorder in 111 (22%), bowel disorders in 214 (42.6%), allergies and dermatological disease in 85 (16.9%), movement dysfunction in 199 (39.6%), and recurrent urinary tract infections in 20 (3.9%).

The prevalence of LUTS is listed in **Table 2**. Urinary incontinence was reported by 12.5% (63/502) of the respondents; it was present only during the day by 22.2% (14/63); only during the night time by 3.8% (19/63); and it was persistent in both the night and day by 47.6% (30/63).

**Table 2:** Prevalence of lower urinary tract symptoms (LUTS) in the autism spectrum disorder (ASD) population.

LUTS	No.	Boys (413)	Girls (89)
Symptomatic patients	387/502 (77.1%)	328/413 (79.4%)	59/89 (66.3%)
Increased frequency	125/502 (24.9%)	108/413 (26.1%)	17/89 (19.1%)
Reduced frequency	41/502 (8.2%)	29/413 (7%)	12/89 (13.5%)
Urgency	151/502 (30.1%)	118/413 (28.6%)	33/89 (37.1%)
Slow stream	147/502 (28.3%)	117/413 (28.3%)	30/89 (33.7%)
Hesitancy	278/502 (55.4%)	225/413 (54.5%)	53/89 (59.5%)
Straining	179/502 (35.6%)	150/413 (36.3%)	29/89 (32.6%)
Voiding symptoms	304/502 (60.5%)	255/413 (61.7%)	49/89 (55%)

The prevalence of incontinence in boys was 10.7% (95% CI: 0.08, 0.14%), while in girls, it was significantly (*p* = 0.006) higher at 21.3% (95% CI: 13.4, 31.3%). On average, the risk of incontinence (**Table 1**) in boys was significantly higher (OR: 9.07; 95% CI: 2.70, 30.43) in individuals with severe ASD compared to those with mild ASD, while it was lower in individuals over 18 years of age (OR: 0.25; 95% CI: 0.08, 0.81) and in the 13–18 age group (OR: 0.29; 95% CI: 0.09, 0.97) compared to patients under 5 years old. As for boys, incontinence was more likely in severe patients in girls (OR: 6.78; 95% CI: 1.37, 33.58), while the age group did not emerge as a possible predictor.

Enuresis was reported by 14.3% (72/502) of the respondents: primary enuresis in 70.8% (51/72), secondary in the remaining. Enuresis was observed in 12.8% of boys (95% CI: 10.0, 16.4%), while in girls, the prevalence was significantly higher (*p* = 0.038) at 21.3% (95% CI: 13.4, 31.3%). As reported in **Table 1**, enuresis in boys showed a strong association with severe disease severity (OR: 8.25; 95% CI: 2.80, 24.39) compared to mild disease severity. Moreover, the likelihood of enuresis decreased progressively with increasing age. Specifically, the odds ratio for the <6-year category decreased from 0.21 (95% CI: 0.08, 0.55) in patients aged 6–12 years to 0.13 (95% CI: 0.05, 0.39) in patients aged 13–18 years, and further to 0.07 (95% CI: 0.02, 0.22) in patients over 18 years. Conversely, in girls, similar to incontinence, enuresis was significantly more likely in patients with severe ASD (OR: 26.95; 95% CI: 4.19, +inf) and did not show any association with age category.

Among boys, 26.4% (95% CI: 22.2, 30.9%) suffered from urinary urgency, while 19.1% (95% CI: 11.5, 28.8%) of girls experienced it. The difference was not statistically significant (*p* = 0.112). No predictors emerged from the logistic regression analysis (**Table 1**), except for a higher probability of experiencing urgency in adult boys (>18 years) compared to those under 6 years old (OR: 0.36; 95% CI: 0.15, 0.87).

Among boys, 26.4% (95% CI: 22.2, 30.9%) suffered from urinary frequency disturbances. Girls had a lower percentage (19.1%; 95% CI: 11.5, 28.8%), although it was not statistically significant (*p* = 0.150). Among boys, the probability of urinary frequency disturbance increased by 2.6 times (OR: 2.6; 95% CI: 1.51, 4.77) in individuals over 18 years old compared to children under 6 years old (**Table 1**). No potential predictors emerged among girls.

In both boys (56.9%; 95% CI: 52.0, 61.7%) and girls (59.6%; 95% CI: 48.6, 69.8%) with ASD, more than half of the subjects experienced alterations in defecation frequency. The difference between the two genders did not reach statistical significance (*p* = 0.647). In boys, this issue was not associated with age or ASD severity (**Table 1**). Conversely, among girls with mild ASD, the problem was observed less frequently (OR: 0.16; 95% CI: 0.04, 0.61) compared to those with severe ASD (OR: 0.18; 95% CI: 0.05, 0.65).

The question evaluating if urine leakage is associated with behavioral disorders demonstrated that incontinence was related to behavioral disorders in 55.5% (35/63) of subjects, and five of them stated that they urinate to try to keep attention, and 30/5 urinate as a sign of a social discomfort.

Pads were used by 40 subjects, with a median of 2.9 pads/day (range, 0–8). Among continent subjects, the use of a pad lasted until the mean age of 3.8 years ± 2.3 years old. A toilet training program was performed by 61 of the respondents, with satisfactory results in 40/61 (65.6%).

Regular bowel function was reported by 57.4% (288/502) of the respondents, while increased daily defecations were present in 11.2% (56/502), constipation in 31.5% (158/502), and fecal incontinence in 7.9% (40/502).

The mean VAS score on the impact of LUTS on family relationships was 2 ± 2.9.

The associations between overall LUTS and ASD severity levels, storage symptoms and ASD severity levels, voiding symptoms, and ASD severity levels are reported in **Tables 3**–**5**, respectively. A positive association between a higher grade of ASD severity and a higher prevalence of LUTS was documented.

**Table 3:** Overall lower urinary tract symptoms (LUTS) and autism spectrum disorder (ASD) levels.

LUTS	ASD Level I (150)	ASD Level II (136)	ASD Level III (216)	*p*-value
Symptomatic patients (pts; 387)	25.3% (98/387)	26.3% (102/387)	48.3% (187/387)	0.01
Pts with LUTS/ASD level pts	65.3% (98/150)	75% (136/150)	86.6% (187/150	0.001

Statistical analysis by ANOVA test.

**Table 4:** Storage lower urinary tract symptoms (LUTS) and autism spectrum disorder (ASD) levels.

Storage LUTS	ASD Level I (150)	ASD Level II (136)	ASD Level III (216)	*p*
**Symptomatic** (258)	27.1% (70/258)	25.6% (66/258)	47.3% (122/258)	0.01
Pts with storage LUTS/ASD level pts	46.6% (70/150)	48.5% (66/136)	56.5% (122/216)	0.01
**Increased daily frequency** (125)	22.4% (23/125)	20.5% (30/125)	57.1% (72/125)	0.01
Pts with increased daily frequency/ASD level pts	18.4% (23/150)	24% (30/136)	57.6% (72/216)	0.01
**Reduced daily frequency** (41)	46.3% (19/41)	35.7% (8/41)	34.1% (14/41)	0.01
Pts reduced daily frequency/ASD level pts	12.6% (19/150)	5.9% (8/136)	5.6% (14/216)	0.01
**Urinary urgency** (151)	23.2% (35/151)	27.8% (42/151)	49% (74/151)	0.01
Pts urinary urgency/ASD level pts	23.3% (35/150)	30.9% (42/136)	34.2% (74/216)	0.01

Statistical analysis by ANOVA test.

**Table 5:** Voiding lower urinary tract symptoms (LUTS) and autism spectrum disorder (ASD) levels.

Voiding LUTS	Level I (150)	Level II (136)	Level III (216)	*p*-value
**Symptomatic** (304)	18.4% (56/304)	26.3% (80/304)	55.3% (168/304)	0.001
Pts with voiding LUTS/ASD level pts	37.7% (56/150)	58.8% (80/136)	77.8% (168/216)	0.001
Slow stream (147)	12.9% (19/147)	23.1% (34/147)	63.9% (94/147)	0.001
Pts with slow stream/ASD level pts	12.6% (19/150)	25% (34/136)	43.5% (94/216)	0.001
Hesitancy (278)	17.6% (49/278)	26.9% (75/278)	55.4% (154/278)	0.001
Pts with hesitancy/ASD level pts	32.7% (49/150)	55.2% (75/136)	71.3% (154/216)	0.001
Straining (179)	18.9% (34/179)	27.9% (50/179)	53.1% (95/179)	0.001
Pts with straining/ASD level pts	22.7% (34/150)	36.8% (50/136)	43.9% (95/216)	0.001

Statistical analysis by ANOVA test.

## Discussion

This survey showed in one of the largest samples that LUTS are very common in young ASD subjects (>70%), particularly voiding symptoms. More than half of the respondents reported storage symptoms, while urinary incontinence and enuresis were less frequent. Urinary incontinence and enuresis were more common in the female population. However, the latter sample was significantly smaller than the male one, and this may have influenced this result. Interestingly, a relationship with ASD severity was found in both genders. Urgency and frequency showed similar rates in both genders and did not appear to be typical of a female or male population. Bowel symptoms were very common in both genders (>50%), highlighting that these are very relevant and typical disorders to be addressed in ASD autism.

LUTS prevalence in young ASD subjects is reported with a wide range, depending on the low sample sizes of the majority of the studies and the objective difficulty of obtaining data on this topic in this population (4, 10–12). Therefore, to date, it is very challenging to uncover the true extent of the problem. This survey demonstrated that LUTS is a pathological condition affecting the vast majority of young ASD subjects. Therefore, these data help to highlight that urological disorders, like other comorbidities, have to be carefully assessed and not ignored or derubricated as a “social problem”. Moreover, due to the very high rate of LUTS in the ASD young population, urologists should be included in the multidisciplinary team of specialists that take care of these subjects. Of course, the exact pathophysiological mechanism underlying LUTS is not clear. In the young ASD population, it is usually assumed that LUTS may be related to behavioral disturbances. Most of the etiopathogenetic mechanisms of voiding dysfunction in the idiopathic ASD population are related to central conditions. A pathophysiological mechanism similar to non-neurogenic neurogenic disorders could sometimes trigger true urinary dysfunction, such as overactive bladder syndrome, underactive bladder syndrome, dyssynergia, or pseudodyssynergia (14). It is difficult, but clinically crucial, to understand if an “organic” and not just functional disorder has occurred with the aid of caregivers, voiding diary, and the use of questionnaires. Therefore, urologists should be advised in cases where LUTS occurs to better assess the severity of the condition and support diagnostic and therapeutic programs, such as toilet training, behavioral therapy, or pharmacological treatments in selected cases. The risk of ignoring a potential chronic condition may leave these people with a low quality of life or expose them to the risk of complications in older age. Therefore, the aim of this study was also to raise awareness of this underestimated condition.

The second endpoint of our study was to assess the potential relationship between LUTS and the severity of ASD. A higher rate of overall LUTS, storage, and voiding symptoms was associated with a great ASD severity. This striking finding could be explained not only by the higher neuropsychiatric disorders but also by greater mobility problems. Both these characteristics may be involved, but the epidemiological nature of this study cannot allow us to fully answer. This is a limitation of our research. However, the “warning” rising from the present study is that in ASD subjects with more severe disorders, urological pathological conditions and LUTS should be carefully evaluated as they can be more common. Interestingly, the negative influence of LUTS on the family relationship was low. This finding may be due to the high awareness and resilience of the family members. Bowel disorders, particularly constipation, were reported by more than half of the respondents. These data showed that in ASD subjects, urinary and bowel symptoms are often associated. A bowel diary might be useful to better gain awareness of a potential pathological condition and to allow clinicians to manage it. A limitation of this study is that most of the data were reported with the aid of caregivers and not by the ASD subject alone. However, this is an intrinsic problem when approaching this population. Another limitation of our study was that the sample size unbalanced by gender, due to a larger male population.

## Conclusions

This survey demonstrated that LUTS are very common in the young ASD population and that urinary symptoms prevalence is related to higher severity of ASD condition. Bowel disorders are often associated with urinary symptoms and dysfunctions. Urologists should be aware of the frequent occurrence of urological disorders and symptoms in individuals with ASD and should be involved in their clinical management in a multidisciplinary team that cares for these people.

## Data availability statement

The original contributions presented in the study are included in the article/**Supplementary Material**. Further inquiries can be directed to the corresponding author.

## Ethics statement

Ethical review and approval was not required in accordance with local and institutional requirements. The participants provided their written informed consent to participate.

## Author contributions

MG, ER, MM, CB, and LZ contributed to the conception and design of the study. ER, MG, and MB organized the database. MG, MB, and GG performed the statistical analysis. ER and MG wrote the first draft of the manuscript. All authors contributed to the article and approved the submitted version.
