# Supplementary material for: National Survey on bladder and bowel dysfunctions in Autism Spectrum Disorder population
Source: Front Psychiatry. 2024 Mar 11;15:1140113. doi: 10.3389/fpsyt.2024.1140113 (PMC10961907; doi:10.3389/fpsyt.2024.1140113)
Supplement: Supplementary file 1 [file DataSheet_1.docx]

Supplementary Material

NATIONAL SURVEY ON BLADDER AND BOWEL DYSFUNCTIONS IN AUTISM SPECTRUM DISORDER POPULATION

**M. Gubbiotti^1^*, M. Balzarro^2^, L. Zoccante^3^, G. Di Gennaro^6^, M. Marchiafava^4^, C. Bedetti^5^, E. Rubilotta^2^**

*** Correspondence:**Marilena Gubbiotti: marilena.gubbiotti@gmail.com

**BBD- A Questionnaire**

**(Bladder & Bowel Dysfunction in Autism- Questionnaire)**

**1. Age** (Age of child- teen/ own age if self-compiled)**:**

**2. Gender (M/F):**

**3. Language:** - Non- verbal

- Verbal, only words

- Verbal, only phrases

- Verbal discourses

**4. Need for support from the family and/or caregiver:**

Mild

Medium

Significant/ Constant

**5. There are fixed repetitive behaviors:**

No/ not very frequent

Quite frequent

Very frequent

**6. There are some of the following disorders?**

Sleep disorders

Bowel disorders

Allergies/ skin

Motor embarrassment

**7. Do you use diaper?**

Yes

No

Only during the night

Only during the day

*7.1* ***If answer is not:***

- Up to how many years did you use the diaper?

*7.2* ***If answer is yes:***

- How many diapers changes in a day?

**8. How often do you urinate during the day:**

Every half an hour

More than 8 times

3- 4 times/ day

1-2 times/ day

**9. Do you sometimes feel an urgency before micturion and that you can't postpone urination?**

Yes

No

**10. Do you have urinary incontinence?**

Yes

No

***10.1 If answer is yes:***

- During the day
- During the night
- Both day and night

**11. Urine leakage is associated with behavioral disorders?**

Yes, micturition is to get attention

Yes, micturition is to show a discomfort

No

**12. Do you have urine leakage during sleep?**

Yes

No

**12.1** ***If answer is yes:***

- It has always been, since childhood
- It reappeared after a period of nocturnal continence

**13. When you need to urinate:**

Go to the bathroom by yourself

Ask to be accompanied

Don't warn and urinate in the diaper

**14. Do you need to urinate in multiple times (flow stops and starts again)?**

Yes

No

Sometimes

A few times

**15. Do you strain your abdomen to urinate?**

Yes

No

Sometimes

A few times

**16. Do you have to wait before micturition begins?**

Yes

No

Sometimes

A few times

**17. Do you have urinary infections?**

Yes

No

***17.1 If answer is yes:***

- Once a month
- Once every 3 months
- 3- 4 times a year

**18. Have you ever followed urination rehabilitation programs (Toilet Training)?**

No

Yes, with poor results

Yes, with good results

**19. How many times do you defecate during the week?**

Everyday

Many times a day

Every 2- 3 days

Every 3- 4 days

**20. Do you have faecal incontinence?**

Ha incontinenza fecale?

Yes

No

Yes, to show a discomfort

**21. How much does your urinary condition affect the family balance (score: 0= not at all; 10= very much)?**
